# Receptor homodimerization significantly prolongs the lifetime of ligand-induced cross-linking of CLEC-2 but not GPVI

**DOI:** 10.1016/j.bvth.2026.100160

**Published:** 2026-03-19

**Authors:** Joanne C. Clark, Eleyna M. Martin, Alexandre Slater, Davide Calebiro, Zsombor Koszegi, Steve P. Watson

**Affiliations:** 1 Department of Cardiovascular Sciences, School of Medical Sciences, College of Medicine and Health, University of Birmingham, Birmingham, United Kingdom; 2 Centre of Membrane Proteins and Receptors, The Universities of Birmingham and Nottingham, Birmingham and Nottingham, United Kingdom; 3 Department of Metabolism and Systems Science, School of Medical Sciences, College of Medicine and Health, University of Birmingham, Birmingham, United Kingdom

## Abstract

The platelet immunoreceptor tyrosine-based activation motif receptors, glycoprotein VI (GPVI) and C-type lectin-like receptor 2 (CLEC-2), are activated by a diversity of ligands that bind to distinct epitopes, indicating that competitive antagonists will not block activation by all stimuli. In this study, we used 2-color single-particle tracking to investigate the dynamic nanoscale organization of the 2 receptors in the cell membrane to identify new strategies for inhibition. The studies were performed in CHO-K1 cells that lack the tyrosine kinase Syk. The results show that, when expressed at low level, GPVI and CLEC-2 diffuse over the cell surface as monomers with the presence of dimers due to random collisions. The addition of divalent and trivalent nanobody ligands induces homodimerization and cessation of movement of both receptors proportionate to ligand valency. The dimers of CLEC-2 are longer-lived than those of GPVI, despite a lower affinity of the monomeric nanobody that forms the ligand backbone. The dimerization of recombinant monomeric CLEC-2 but not GPVI was detected by surface plasmon resonance with an affinity constant of 18.5μM. The results suggest that the prolonged lifetime of the CLEC-2 interactions is due to synergy between ligand-induced cross-linking and receptor homodimerization. Blocking dimerization may be an effective way to inhibit the activation of CLEC-2 by its diverse range of ligands.

## Key Points

• GPVI and CLEC-2 are monomers and undergo homodimerization and immobilization upon ligand addition, thereby supporting clustering.
• CLEC-2 dimer lifetime is greater than that of GPVI, indicating synergy between homodimerization and ligand-induced dimerization of CLEC-2.

## Visual Abstract

**Figure:**
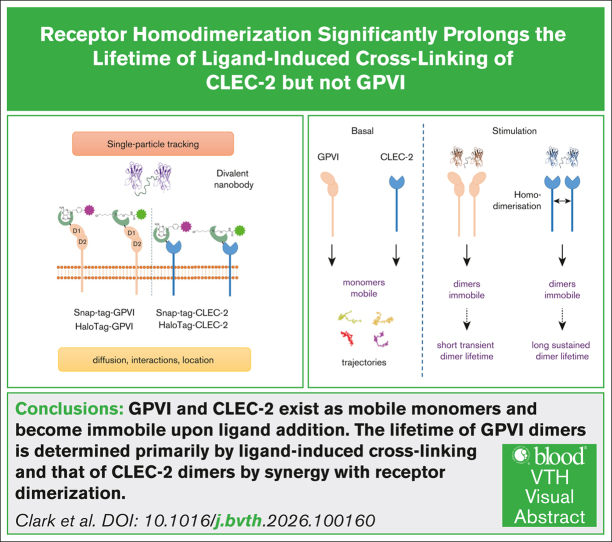

## Introduction

Platelets have a fundamental role in the control of hemostasis and thrombosis and additional roles in inflammation and host defense.^1^ Current antiplatelet therapy for the treatment and prevention of cardiovascular disease pathophysiology targets G protein–coupled receptors including dual antiplatelet therapy (aspirin and a P2Y_12_ antagonist). However, this treatment increases the risk of life-threatening bleeding, is largely ineffective in inflammation-driven thrombosis (known as thromboinflammation), and is insufficient in preventing reoccurrence.^1^, ^2^, ^3^ Therefore, there is an unmet need for new, more powerful antiplatelets for the treatment and prevention of thrombosis while preserving hemostasis. The platelet tyrosine kinase–linked receptors glycoprotein VI (GPVI) and C-type lectin-like receptor 2 (CLEC-2) have critical roles in thrombosis and thromboinflammation but minimal roles in bleeding,^4^, ^5^, ^6^, ^7^, ^8^, ^9^, ^10^, ^11^, ^12^, ^13^ making them potential ideal targets.

GPVI is a member of the immunoglobulin receptor superfamily^14^ and has numerous endogenous and exogenous ligands, including collagen, fibrin(ogen), snake toxins (eg, convulxin), and diesel exhaust particles.^14^^,^^15^ GPVI has 2 extracellular immunoglobulin domains (D1 and D2), a mucin-rich stalk, a single transmembrane helix, and a cytoplasmic tail. It is noncovalently associated with the Fc receptor γ-chain (FcRγ).^14^^,^^16^^,^^17^ CLEC-2 has an extracellular C-type lectin-like domain, a single transmembrane region, and a cytoplasmic tail.^18^^,^^19^ Its major endogenous ligand is the transmembrane protein podoplanin; in addition, it is activated by heme, the snake toxin rhodocytin, and diesel exhaust particles.^15^ GPVI signals through an immunoreceptor tyrosine-based activation motif (ITAM) in the FcRγ homodimer defined by 2 YxxL (single amino acid code).^14^^,^^16^^,^^17^ In contrast, CLEC-2 signals through a single YxxL in its cytosolic tail, known as a hemITAM.^18^, ^19^, ^20^ The phosphorylation of the conserved tyrosines is initiated by clustering and leads to the binding of Syk via its tandem SH2 domains and the activation of a signaling cascade involving LAT and PLCγ2.^14^^,^^16^, ^17^, ^18^, ^19^, ^20^

The study of the spatial-temporal organization of the receptors is critical to understanding the events that lead to activation. We have previously shown that both receptors are predominantly monomeric when expressed at low level, with dimerization in the case of CLEC-2 increasing in proportion to expression as expected for a low affinity, reversible association.^21^^,^^22^ In line with this, the dimerization of the recombinant Fc-C–type lectin-like domains of human and mouse CLEC-2 has been shown by surface plasmon resonance (SPR) spectroscopy with affinity constant (K_D_) of 278nM and 499nM, respectively.^23^ In contrast, the immunoglobulin domains of GPVI are monomeric at concentrations up to 100μM when measured by analytical ultracentrifugation, with the dimerization of this region only detected in crystal structures.^24^^,^^25^

In this study, we investigate the dynamic nanoscale organization of GPVI and CLEC-2 using 2-color single-particle tracking (SPT) under basal conditions and following stimulation with multivalent nanobodies of known stoichiometry. The studies were performed in Chinese hamster ovary K1 (CHO-K1) cells whose relatively flat structures facilitate the tracking of individual fluorophores. Furthermore, they do not express the tyrosine kinase Syk, which enables the study of the interaction of between ligand-induced cross-linking and receptor dimerization.

The results show that GPVI and CLEC-2 are monomeric and predominantly mobile when expressed at a low level. The addition of multivalent nanobody ligands leads to receptor homodimerization and a reduction in movement in proportion to their valency. The lifetime of CLEC-2 homodimers is prolonged relative to that of GPVI dimers despite a lower affinity of the nanobody that forms the backbone of the multivalent nanobodies, indicating synergy with receptor dimerization.

## Methods

The full description of the materials and methods can be found in the supplemental Methods.

### GPVI and CLEC-2 nanobodies

Nanobodies targeted against the extracellular domain of GPVI and CLEC-2 were generated and cross-linked as previously reported.^22^^,^^26^^,^^27^

### SPT

SPT was performed using total internal reflection fluorescence illumination on a custom system.^28^ Automated single-particle detection and tracking were performed with the u-track software,^29^ and the obtained trajectories were further analyzed using custom algorithms in the MATrix LABoratory (MATLAB) environment.^28^^,^^30^

### Statistical analysis

Results are shown as mean ± standard deviation unless otherwise stated. The number of independent experiments is described in the figure legends. Data were analyzed using PRISM version 10.4.0 (GraphPad, San Diego, CA). Deconvolution curves were fitted using MATLAB 2024b. Data were tested for normality using the Shapiro-Wilk test. For diffusion groups, a 2-way analysis of variance with a Tukey post hoc test was used. For NFAT reporter assays and k_on_ measurements, the Student 2-tailed unpaired *t* tests were used. Significance was set at *P* ≤ .05.

## Results

### SNAP-tag and HaloTag GPVI and CLEC-2 are functional

**Figure 1.**
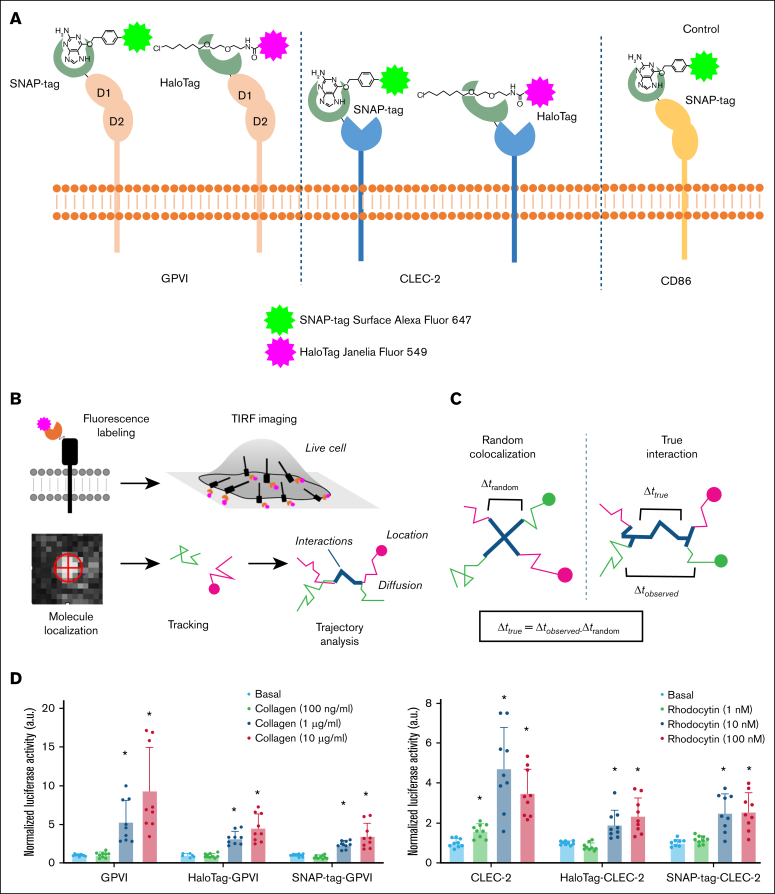
**GPVI and CLEC-2 constructs used for SPT experiments are functional.** (A) Schematic of GPVI and CLEC-2 receptors tagged at the extracellular region with a SNAP- or HaloTag labeled with Alexa Fluor-647 and Janelia Fluor-549, respectively. (B) Schematic of the SPT imaging and analysis strategy. Tagged receptors are labeled with fluorescent dyes and are expressed and imaged in live cells using fast multicolor total internal reflection fluorescence (TIRF) microscopy. Molecules are localized and tracked to generate trajectories that can be analyzed to provide information on location, diffusion, and interactions. (C) Schematic showing the comparison between random colocalizations and true interactions. The observed duration of interactions (Δ*t*_observed_) corresponds to their true duration (Δ*t*_true_) plus that of random colocalizations (Δ*t*_random_). The distribution of true durations is estimated by deconvolution. (D) DT40 chicken B cells were transfected with an NFAT-luciferase receptor construct and 2 μg of wild-type GPVI or CLEC-2 and SNAP-tag and HaloTag GPVI or CLEC-2 constructs. For GPVI samples, 2 μg of FcRγ chain was also cotransfected. Cells were unstimulated or stimulated for 6 hours and then lysed and assayed for luciferase activity. Luciferase activity normalized for basal values, unstimulated and stimulated with collagen (100 ng/mL and 1 and 10 μg/mL) for GPVI or rhodocytin (1nM, 10nM, and 100nM) for CLEC-2. Each experiment was performed in triplicate. Significance was measured with the Student 2-tailed *t* test where *P* ≤ .05 is denoted by an asterisk. All data are presented as mean ± standard deviation (SD; n = 3 independent experiments).

To investigate the dynamics of homodimerization of GPVI and CLEC-2 on the membrane of living cells with high spatial (∼20 nm) and temporal (∼30 milliseconds) resolution,^30^, ^31^, ^32^ the receptors were labeled via extracellular SNAP or Halo tags (Figure 1A) and transiently coexpressed in CHO-K1 cells at ∼10 times lower than their level in platelets (supplemental Figure 1A) to facilitate visualization of individual receptors. In addition, HaloTag-GPVI or HaloTag–CLEC-2 was transiently coexpressed with the monomeric protein CD86 labeled with SNAP-tag to correct for random colocalizations.^30^ The transfected receptors were labeled with saturating concentrations of SNAP-Surface Alexa Fluor-647 and HaloTag Janelia Fluor-549 for simultaneous imaging using fast 2-color single-molecule total internal reflection fluorescence microscopy in combination with SPT (Figure 1A-B).^30^^,^^31^ Fluorophores are localized and tracked to generate trajectories that can be analyzed to provide information on receptor location, diffusion, and interactions (Figure 1B). True interactions were separated from random colocalizations as described in Figure 1C.^30^ The observed duration of interactions (Δ*t*_observation_) corresponds to their true duration (Δ*t*_true_) plus that of random colocalizations (Δ*t*_random_). The distribution of true durations can be estimated by deconvolution. The functionality of the tagged receptors was shown (Figure 1D) using an NFAT reporter assay.^33^

It is important to emphasize that the analysis is based on 2-color imaging, measured using 2 different fluorophores. Therefore, although a receptor trimer could consist of a dimer of 1 color and monomer of the other, our analysis would only detect the interaction between the 2 different colors and not recognize the “same color” dimer.

### SPT shows that GPVI becomes immobile upon cross-linking

**Figure 2.**
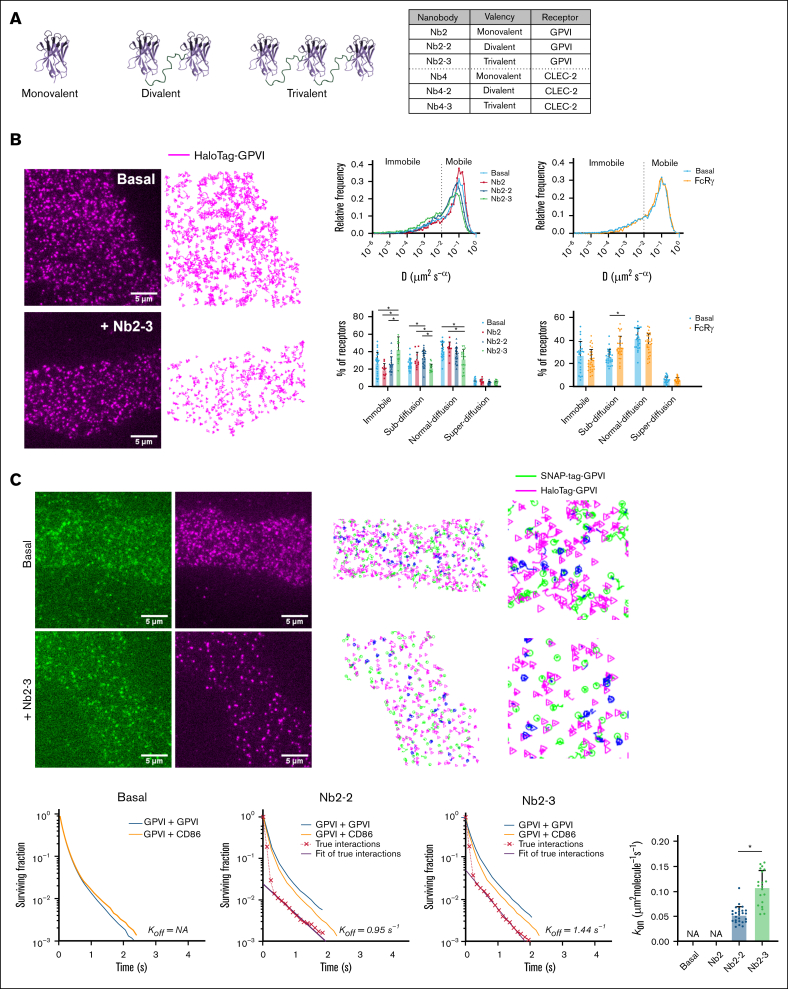
**GPVI is predominantly monomeric under basal conditions and forms homodimers and becomes immobile on ligand addition.** (A) Schematic representation of the monovalent, divalent, and trivalent GPVI and CLEC-2 nanobody ligands. The table shows the nomenclature and valency of the ligands. (B-C) TIRF microscopy was used to image live CHO cells for 400 frames (∼12 seconds) expressing the receptor combinations: HaloTag-GPVI + SNAP-tag–GPVI and HaloTag-GPVI + SNAP-tag–CD86. CD86 was used as a noninteracting control protein. Generated videos were tracked to generate trajectories and analyzed as described in “Methods.” (B) Single-color SPT was used to analyze the diffusion of GPVI receptors. Representative images of a CHO cell imaged using TIRF microscopy under basal conditions and following stimulation with trivalent Nb2-3 (100nM) where magenta = HaloTag-GPVI labeled with Janelia Fluor-549 and the corresponding tracking analysis showing the trajectories of HaloTag-GPVI. An analysis of the trajectories was used to generate diffusion coefficients (*D*) of HaloTag-GPVI. The proportion of receptors that were mobile (*D* ≥ 0.01 μm^2^/s^-α^) or immobile (*D* < 0.01 μm^2^/s^-α^) was determined. Trajectories were classified into 4 diffusion groups: immobile, normal diffusion (Brownian movement), subdiffusion (confined movement), and superdiffusion (directional movement) according to *D* and anomalous diffusion exponent (α) (see “Methods”). (C) Two-color SPT was used to analyze GPVI-GPVI interactions under basal conditions and following ligand addition. Representative images of a CHO cell imaged using TIRF microscopy under basal conditions and following stimulation with trivalent Nb2-3 (100nM) where green = SNAP-tag–GPVI labeled with Alexa Fluor-647 and magenta = HaloTag-GPVI labeled with Janelia Fluor-549; the corresponding dual-channel tracking analysis show the trajectories of SNAP- and HaloTag-GPVI. Blue shows the colocalization of the 2 channels (scale bar, 5 μm). Interaction survival curves for GPVI-GPVI (blue) and GPVI-CD86 (orange) interactions under basal condition and following divalent (Nb2-2, 100nM) and trivalent (Nb2-3, 100nM) stimulation. Calculated true interaction curves based on deconvolution are shown in red and fitted (black) to give the rates of association (K_on_) and dissociation (K_off_) of receptor interactions. For K_on_, each data point represents a cell. Significance was measured with (panel B) 2-way analysis of variance (ANOVA) with a Tukey post hoc test and (panel C) the Student 2-tailed *t* test where *P* ≤ .05 is denoted by an asterisk. Data are presented as mean ± SD (n = 6 independent experiments). Number of cells: GPVI-GPVI basal = 27, GPVI-CD86 = 9, Nb2 = 11, Nb2-2 = 26, Nb2-3 = 20, and FcRγ = 33. NA, not applicable (as no dimerization was detected).

Single-color SPT was used to analyze the movement of GPVI receptors. Recordings were conducted at a rate of 1 image every 30 milliseconds, with each recording limited to 12 seconds to minimize the impact of photobleaching. We utilized divalent (Nb2-2) and trivalent (Nb2-3) nanobodies, derived from a monovalent nanobody against GPVI, Nb2, to induce activation (Figure 2A).^34^ The multivalent nanobodies have a higher affinity for GPVI than monovalent Nb2 (K_D_ = 0.58nM) due to avidity.^34^

The individual trajectories of HaloTag-GPVI receptors were evaluated by mean square displacement analysis to determine diffusion coefficients under basal conditions (Figure 2B).^30^ Trajectories were classified into 4 groups: immobile, normal diffusion (Brownian movement), subdiffusion (confined movement), and superdiffusion (directional movement) according to the estimated diffusion coefficient (D) and anomalous diffusion exponent (α).^30^ Over the period of recording, 27% of GPVI was immobile, 26% confined, and 42% showed Brownian motion, with <6% showing directional motion (Figure 2B). Similar results were observed in CHO-K1 cells cotransfected with GPVI and FcRγ, with an ∼10% increase in confined movement and a nonsignificant reduction in normal diffusion (Figure 2B). The increase in confinement may reflect a reduction in the rate of movement of the larger particles (Brownian motion is inversely proportional to particle size) or trapping in small cytoskeletal compartments.

We next sought to investigate the dynamics of movement of GPVI at the plasma membrane upon stimulation. The divalent and trivalent nanobody ligands induced a rapid increase in the immobile fraction that could be observed visually and was similar in recordings conducted in the first 15 minutes or between 15 and 30 minutes (Figure 2B and data not shown). A significant increase in the immobile fraction was observed with Nb2-3, increasing from 27% to 42% (*P* < .05), but not with Nb2-2, relative to Nb2, which had no significant effect on the mobility of GPVI (Figure 2B).

These results show that GPVI is predominantly mobile under basal conditions, with a subset of receptors being immobile. The immobile fraction increases upon ligand addition in proportion to ligand valency.

### GPVI is monomeric under basal conditions and forms homodimers upon ligand addition

Two-channel SPT imaging of SNAP-tag and HaloTag-GPVI was used to measure the stoichiometry of GPVI using SNAP-tag–CD86 as a control for random collisions (Figures 1C and 2C; supplemental Video 1). The 2 tagged forms of GPVI did not colocalize for longer times than measured between SNAP-tag–CD86 and HaloTag-GPVI indicating that only random collisions were occurring between GPVI receptors, which are therefore monomeric (Figure 2C). A similar result was observed in the presence of FcRγ (supplemental Figure 2A-B).

We next investigated the effect of the divalent and trivalent nanobodies on GPVI-GPVI interactions (Figure 2C; supplemental Figure 2A; supplemental Videos 2 and 3). The interaction survival curves for GPVI in the presence of Nb2-3 were shifted to the right compared with the CD86 curves, indicating that specific interactions were occurring alongside the random collisions (Figure 2C). A similar result was observed with Nb2-2 but with a reduced number of specific interactions (Figure 2C). The difference between the 2 ligands can be accounted by an increased level of clustering attributed to the greater valency.

The results show that GPVI is monomeric at this level of expression and that multivalent ligands stimulate the formation of dimers, with the response proportional to valency.

### SPT shows that CLEC-2 becomes immobile upon cross-linking

**Figure 3.**
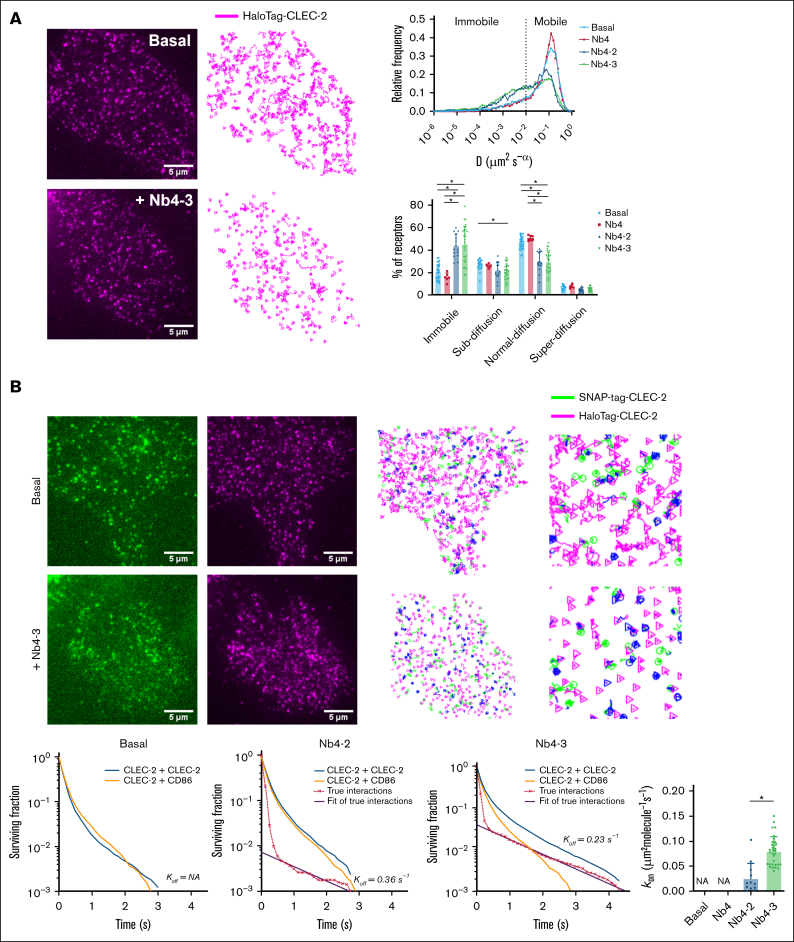
**CLEC-2 is predominantly monomeric under basal conditions and forms homodimers upon ligand addition.** (A-B) TIRF microscopy was used to image live CHO cells for 400 frames (∼12 seconds) expressing the receptor combinations: HaloTag-CLEC-2 + SNAP-tag–CLEC-2 and HaloTag–CLEC-2 + SNAP-tag–CD86. Generated videos were tracked to generate trajectories and analyzed as described in “Methods.” (A) Single-color SPT was used to analyze the diffusion of CLEC-2 receptors. Representative images of a CHO cell imaged using TIRF microscopy under basal conditions and following stimulation with trivalent Nb4-3 (100nM): magenta = HaloTag–CLEC-2 labeled with Janelia Fluor-549; corresponding tracking analysis showing the trajectories of HaloTag–CLEC-2. An analysis of the trajectories was used to generate diffusion coefficients (*D*) of HaloTag–CLEC-2 under basal conditions and following ligand addition: Nb4 (100nM), divalent Nb4-2 (100nM), and trivalent Nb4-3 (100nM). The proportion of receptors that were mobile (*D* ≥ 0.01 μm^2^/s^-α^) or immobile (*D* < 0.01 μm^2^/s^-α^) was determined. For other details, see Figure 2. (B) Two-color SPT was used to analyze CLEC-2–CLEC-2 interactions. Representative images of a CHO cell imaged using TIRF microscopy under basal conditions and following stimulation with trivalent Nb4-3 (100nM) where green = SNAP-tag–CLEC-2 labeled with Alexa Fluor-647 and magenta = HaloTag-CLEC-2 labeled with Janelia Fluor-549 The corresponding dual-channel tracking analysis show the trajectories of SNAP-tag and HaloTag–CLEC-2. Blue shows colocalization of the 2 channels (scale bar, 5 μm). Interaction survival curves for CLEC-2–CLEC-2 (blue) and CLEC-2–CD86 (orange) interactions under basal condition and in the presence of divalent (Nb4-2, 100nM) and trivalent (Nb4-3, 100nM) ligands. Calculated true interaction curves based on deconvolution are shown in red and fitted (black) to give the rates of association (K_on_) and dissociation (K_off_) of interactions. For K_on_, each data point represents a cell. Significance was measured with (panel A) 2-way ANOVA with a Tukey post hoc test and (panel B) the Student 2-tailed *t* test where *P* ≤ .05 is denoted by an asterisk. Data are presented as mean ± SD (n = 6 independent experiments). Number of cells: CLEC-2–CLEC-2 basal = 33, CLEC-2–CD86 = 15, Nb4 = 6, Nb4-2 = 15, and Nb4-3 = 30. NA, not applicable (as no dimerization was detected).

SPT measurements were used to measure the movement of CLEC-2 (Figure 3A) in the presence of divalent (Nb4-2) and trivalent (Nb4-3) nanobodies derived from the monovalent nanobody, Nb4 (also known as LUAS).^22^^,^^34^ The K_D_ of Nb4 for CLEC-2 is 137nM.^34^

We monitored the individual trajectories of CLEC-2 as described earlier.^31^ The proportions of CLEC-2 that were immobile, confined, or showing Brownian motion were 20%, 27%, and 46%, respectively, with <7% of receptors showing directional motion (Figure 3A). These results show that most CLEC-2 receptors are mobile over the 12 seconds of recording.

We next sought to investigate the dynamics of movement of CLEC-2 in the presence of Nb4-2 and Nb4-3. As was the case with GPVI, the addition of the multivalent ligands led to a rapid increase in the immobile fraction of CLEC-2, which could be observed visually (Figure 3A). Measurements conducted in the first 15 minutes or between 15 and 30 minutes showed a similar increase in the immobile fraction, demonstrating that the change in movement was sustained (Figure 3A and data not shown). Nb4-2 and Nb4-3 caused a significant increase in the immobile fraction from 20% to 44% and 45%, respectively, relative to Nb4 (*P* < .05), which had no significant effect on the mobility of CLEC-2 (Figure 3A). There was also a significant difference between the 2 divalent ligands for GPVI and CLEC-2 (*P* < .05), but not between the trivalent ligands.

These results show that CLEC-2 is predominantly mobile under basal conditions, with a subset of receptors being immobile and increasing in the presence of the divalent and trivalent nanobody ligands.

### CLEC-2 is predominantly monomeric and forms homodimers upon cross-linking

As with GPVI, 2-channel SPT imaging was used to measure the homodimerization of CLEC-2 using SNAP-tag and HaloTag–CLEC-2 receptors. The frequency and duration of the specific CLEC-2–CLEC-2 interactions were calculated by the deconvolution of the apparent colocalization times with those of random colocalizations estimated using CD86 as for GPVI (Figure 1C).^30^ Under basal conditions, SNAP- and Halo-tagged CLEC-2 receptors did not colocalize for longer than SNAP-tag–CD86 and HaloTag–CLEC-2, indicating that only random collisions are occurring between the differentially tagged CLEC-2 receptors (Figure 3B; supplemental Video 4). Therefore, CLEC-2 is monomeric at this level of expression (Figure 3B). However, in the presence of the trivalent ligand, Nb4-3, the interaction survival curves for CLEC-2 were shifted to the right and prolonged compared with the CD86 curve, indicating a specific interaction; a similar result was observed upon the addition of Nb4-2 but with a reduced effect (Figure 3B; supplemental Videos 5 and 6).

These results show that CLEC-2 is monomeric under basal conditions and forms homodimers in the presence of the divalent and trivalent nanobodies, with the increase in proportion to ligand valency.

### CLEC-2 homodimer lifetime is prolonged compared with that of GPVI

We next compared the kinetics of the interactions of GPVI and CLEC-2 by estimating the association and dissociation rates and the duration of the interaction. The addition of Nb2-2 and Nb2-3 resulted in an increase in the on-rate of dimerization (k_on_) of GPVI of ∼0.05 and 0.12 molecules per μm^2^ per second, respectively (Figure 2C). In comparison, the addition of Nb4-2 and Nb4-3 resulted in a lower increase in the k_on_ of CLEC-2 dimerization of 0.03 and 0.08 molecules per μm^2^ per second, respectively (Figure 3B), which is explained by the slower on-rate of association of the monovalent CLEC-2 nanobody.^34^ For both receptors, the trivalent ligand caused a slightly faster k_on_, demonstrating the importance of ligand avidity in the GPVI-GPVI and CLEC-2–CLEC-2 interactions.

We used the true interaction survival curves for GPVI (Figure 2C) and CLEC-2 (Figure 3B) to estimate the rate of dissociation (k_off_) following treatment with the nanobody ligands. The GPVI-GPVI interaction showed a more rapid rate of dissociation than that for CLEC-2–CLEC-2 in the presence of trivalent ligands despite the higher affinity and slower dissociation rate of monovalent Nb2 relative to monovalent Nb4.^34^ In the presence of Nb2-3, the k_off_ of GPVI was 1.44 per second (95% confidence interval, 1.06-1.83) compared with that of 0.23 per second (95% confidence interval, 0.16-0.30) for CLEC-2 in the presence of Nb4-3 (Figures 2C and 3C). This equates to a longer dimer lifetime for CLEC-2 of 4.3 seconds compared with that for GPVI of 0.70 second in the presence of the trivalent ligands. A similar difference was observed between the divalent nanobodies for CLEC-2 and GPVI, with the lifetime for CLEC-2 in the presence of Nb4-2 of 2.7 seconds, which is longer that for GPVI in the presence of the trivalent nanobody ligand.

These results show that the CLEC-2 interaction induced by the corresponding multivalent nanobodies is more prolonged than for GPVI despite the lower affinity and faster off-rate of dissociation of the parent nanobodies that form their backbone. This difference can be explained by the ability of the C-type lectin-like domain in CLEC-2 to dimerize, whereas this is not the case for the immunoglobulin domains in GPVI.^23^^,^^25^

### SPR measurements of GPVI and CLEC-2

**Figure 4.**
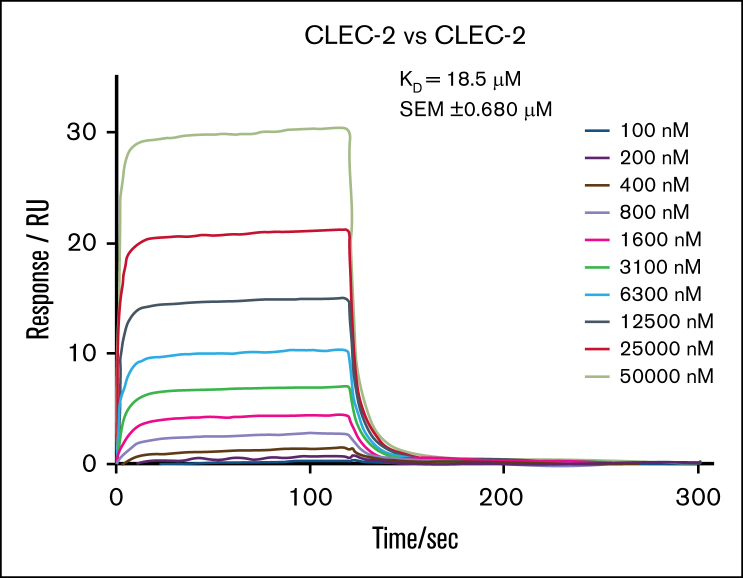
**The monomeric C-type lectin-like domain of CLEC-2 dimerizes in solution using SPR.** A representative SPR sensorgram showing the binding of monovalent C-type lectin-like domain of CLEC-2 to an immobilized surface of itself (extracellular domain residues 55-229). The binding affinity, K_D_, was 18.5μM ± 0.7μM. Values were calculated by kinetic analysis using a global fitting model within the Biacore T200 evaluation software. Data are presented as mean ± SEM (n = 3 independent experiments). RU, resonance units; SEM, standard error of the mean.

As discussed earlier, the homodimerization of recombinant Fc constructs of human and mouse C-type lectin-like domains has been shown by SPR, with K_D_ values of 278nM and 499nM, respectively.^23^ However, these values represent the net product of the affinity of binding of the monomeric protein and avidity due to the presence of 2 copies in the Fc construct. Applying the same methodology to the monomeric C-type lectin-like domain in CLEC-2 revealed an ∼50-fold reduction in affinity, with a K_D_ of 18.5μM ± 0.7μM (Figure 4), reflecting the loss of avidity. In contrast, the dimerization of the recombinant immunoglobulin domains of GPVI was not observed at concentrations up to 20μM, in agreement with measurements using analytical ultracentrifugation.^25^

## Discussion

This study was conducted to investigate the nanoscale organization and dynamics of GPVI and CLEC-2 and to increase our understanding of the mechanisms of dimerization and higher-order clustering of the 2 receptors to identify new mechanisms of inhibition. The results were as follows: (1) at expression levels below those found in platelets, GPVI and CLEC-2 are primarily monomeric and mobile, with colocalizations due to random collisions; (2) divalent and trivalent nanobodies to both receptors stimulate the formation of homodimers and cause an increase in the proportions of confined and immobile receptors; (3) despite the lower affinity of the parent nanobody to CLEC-2 relative to that for GPVI (137nM vs 0.58nM, respectively),^3^ the lifetime of the CLEC-2–CLEC-2 interactions in the presence of the trivalent ligands are prolonged to greater than sixfold relative to those for GPVI-GPVI, with a similar difference observed for the divalent ligands to the 2 receptors; (4) the coexpression of GPVI with FcRγ causes a small increase in confinement but does not induce the formation of dimers; and (5) recombinant monomeric CLEC-2 but not GPVI dimerizes with a K_D_ of 18.5μM ± 0.7μM. These results suggest that the lifetime of dimers of CLEC-2 is regulated through synergy between ligand-induced cross-linking and receptor homodimerization in the CHO-K1 cells, which lack the tyrosine kinase Syk. This extends our previous observation in HEK293T cells that the clustering of CLEC-2 is supported by cross-linking of phosphorylated cytosolic tails by Syk, with all 3 mechanisms acting in synergy as shown using agent-based modeling.^26^

The absence of dimerization of GPVI under basal conditions and the conclusion that this plays a minimal role in supporting clustering is in line with biochemical measurements using the recombinant immunoglobulin domains of GPVI. The dimerization of GPVI was not detected by ultracentrifugation^24^ or SPR (this study) at concentrations of up to 100μM. The remaining extracellular portion of GPVI is a heavily O-glycosylated stalk that is predicted to repel dimerization. The presence of dimers of the immunoglobulin domains in the crystals of GPVI is therefore likely to be due to packing as originally proposed.^24^

Our results bring to a close the debate on whether a significant proportion of GPVI is expressed as a dimer and whether the dimeric form adopts a conformation that is required for the interaction with collagen.^35^, ^36^, ^37^, ^38^, ^39^ The latter was shown by the demonstration that the X-ray crystal structures of the monomeric and dimeric forms of GPVI are the same and that collagen binds to the first immunoglobulin domain in GPVI, distinct from the proposed site of dimerization in the second immunoglobulin domain.^27^^,^^39^, ^40^, ^41^, ^42^, ^43^ However, our observations also refute other lines of evidence of significant dimerization of GPVI, such as those made using the coprecipitation of differentially tagged receptors or bioluminescence resonance energy transfer measurements.^21^^,^^44^^,^^45^ These studies were nonquantitative and used high levels of receptor expression, increasing the incidence of random collisions and nonspecific interactions and potentially giving rise to these observations. Furthermore, our findings have significant implications for the mode of inhibition of the GPVI-blocking Fab, glenzocimab, which has been shown to bind at the site of dimerization in the GPVI crystals.^41^ The inhibitory activity of glenzocimab is primarily mediated by the inhibition of binding of collagen because of steric hindrance.

The apparent absence of dimerization of CLEC-2 under basal conditions in the CHO-K1 cells in this study is in line with the affinity of homodimerization (K_D_ = 18.5μM) and the low level of expression, which is ∼10% of that in human platelets; the concentration of CLEC-2 in the membrane of human platelets has been estimated to be 2.2μM.^26^ Although it is possible that homodimeric interactions did occur at an extremely low level, the detection method may not have been sensitive enough to detect such short-lived interactions, consistent with the rapid rate of dissociation of the CLEC-2–CLEC-2 interaction. Moreover, the affinity of homodimerization indicates that CLEC-2 is also likely to be primarily monomeric in platelets. Conversely, in mouse platelets, which express an ∼10-fold higher level of CLEC-2, a significant fraction (>50%) of CLEC-2 is predicted to be dimeric, explaining why divalent ligands activate mouse but not human platelets.^22^

Although CLEC-2 is expressed as a monomer, our results show that its ability to dimerize plays a significant role in extending the lifetime of ligand-induced cross-linking of the C-type lectin-like receptor relative to that of GPVI by the multivalent nanobodies. This difference in the regulation of clustering between the 2 receptors may be related to the number of YxxLs in their cytosolic tails. CLEC-2 has a single YxxL (hemITAM) and therefore requires 2 phosphorylated receptors for the recruitment and activation of Syk.^18^ In contrast, GPVI has 4 YxxLs present in the 2 ITAMs in the associated FcRγ homodimer. The inability of GPVI to dimerize may help to limit activation under basal conditions.

The observation that both receptors become immobile in the presence of multivalent ligands has been observed for other receptors, including the μ-opioid G protein–coupled receptor^46^ and the MET tyrosine kinase receptor, with dimerization resulting in a reduction in the rate of diffusion.^47^ A significant reduction in the rate of diffusion of CLEC-2 upon ligand engagement was not observed, possibly due to the limits of detection of the current methodology. Although ligand engagement is predicted to reduce the rate of Brownian motion in proportion to the inverse of the particle size, it should not result in a loss of movement. The reduced mobility could therefore be due to confinement within small cytoskeletal compartments,^48^ with monomers escaping more easily than oligomers, or recruitment to cholesterol-rich domains, although there is no direct evidence for either mechanism. However, it is unlikely to be due to downstream signaling events because Syk is not expressed in CHO-K1 cells. The change in movement could lead to an alteration in phosphorylation of the (hem)ITAM receptors or, in platelets, the extent and duration of recruitment of Syk and downstream signaling.

It is important to consider the significance of our results to the biological and pathological roles of GPVI and CLEC-2. Both receptors have a diverse range of ligands that either have multiple epitopes or, in the case of membrane proteins, are restricted on the cell surface. The diverse range of ligands bind to differing sites in the 2 receptors, and with varying affinities, but have in common the ability to induce clustering to initiate downstream signaling. For ligands that bind solely to either receptor, such as collagen-related peptide or podoplanin, the nanobodies can be considered an effective mimetic with the key experimental advantage of a known stoichiometry that facilitates the analysis of the experimental data.^34^ In contrast, they cannot be considered to be an effective mimic of ligands that bind to >1 receptor such as collagen (GPVI and integrin α2β1), fibrin (GPVI and integrin αIIbβ3), heme, and diesel exhaust particles (GPVI and CLEC-2) because the binding to the second receptor will increase affinity due to avidity and potentially contribute to downstream signaling. These considerations also apply to other ITAM receptors such as the low-affinity immune receptor FcγRIIA, which has a single ITAM in its cytosolic chain and serves as a receptor for immune complexes, and the B-cell antigen receptor, which is composed of a membrane-bound immunoglobulin molecule and a disulfide-linked heterodimer, immunoglobulin-α/immunoglobulin-β, with each copy containing a single ITAM. Although the ligand for FcγRIIA is monomeric, several models have been proposed on how antigen binding to the B-cell receptor induces activation, including conformational change and receptor clustering.^49^

Previously, we have used agent-based modeling to show how the synergy between receptor homodimerization and cross-linking of phosphorylated hemITAMs by Syk contributes to the extent and duration of clustering of CLEC-2 in the presence of a divalent ligand.^26^ In this study, we have shown that homodimerization alone can also do this. However, the importance of each of these mechanisms to clustering to platelets and the degree to which this varies between ligands are unclear.

A limitation of this study is the use of a transfected cell line to achieve stoichiometric labeling of GPVI and CLEC-2 and the low level of expression. The same methodology cannot be replicated in platelets due to their anucleate nature and the level of expression that hampers the visualization of individual proteins. Therefore, the development of stoichiometrically labeled high-affinity probes is critical.

In conclusion, this study demonstrates that GPVI and CLEC-2 are monomeric when expressed at low levels and mobile, with ligand engagement inducing the formation of homodimers and a reduction in mobility. Our results show a fundamental difference in the mode of clustering of GPVI and CLEC-2, with the former governed primarily by the ligand affinity/valency and the latter through synergy between ligand engagement and receptor homodimerization. This difference highlights dimerization as a potential target for the inhibition of CLEC-2 but not of GPVI, with the advantage over competitive inhibitors that will inhibit activation by ligands that bind to distinct epitopes. Considering that the dimerization of CLEC-2 is dependent on glycosylation,^50^ this could be targeted by deglycosylation or by an inhibitor. The development of the latter will require the mapping of the site of dimerization.

Conflict-of-interest disclosure: S.P.W. and A.S. have a patent for the anti–glycoprotein VI nanobody Nb2 (WO2022/136457). The remaining authors declare no competing financial interests.

## Supplementary data

Supplemental Methods and Figure

Supplemental Video 1 left side

Supplemental Video 1 right side

Supplemental Video 2 left side

Supplemental Video 2 right side

Supplemental Video 3 left side

Supplemental Video 3 right side

Supplemental Video 4 left side

Supplemental Video 4 right side

Supplemental Video 5 left side

Supplemental Video 5 right side

Supplemental Video 6 left side

Supplemental Video 6 right side
