# Supplementary material for: Receptor homodimerization significantly prolongs the lifetime of ligand-induced cross-linking of CLEC-2 but not GPVI
Source: Blood Vessel Thromb Hemost. 2026 Mar 19;3(2):100160. doi: 10.1016/j.bvth.2026.100160 (PMC13181292; doi:10.1016/j.bvth.2026.100160)
Supplement: Supplemental Methods and Figure [file BVTH_VTH-2025-000487-mmc1.pdf]

Supplementary Materials

Receptor homodimerisation significantly prolongs the lifetime of ligand-induced crosslinking of CLEC-2 but not GPVI

Joanne C. Clark[1,2*], Eleyna M. Martin[1,2], Alexandre Slater[1,2], Davide Calebiro[2,3], Zsombor Koszegi[2,3,*] Steve P. Watson[1,2]

[1]Department of Cardiovascular Sciences, School of Medical Sciences, College of Medicine and Health, University of Birmingham, Edgbaston, Birmingham, B15 2TT, UK.

[2]Centre of Membrane Proteins and Receptors (COMPARE), The Universities of Birmingham and Nottingham, The Midlands, UK.

[3]Department of Metabolism and Systems Science, School of Medical Sciences, College of Medicine and Health, University of Birmingham, Edgbaston, Birmingham, B15 2TT, UK.

***Corresponding authors:** Dr. Joanne C Clark[1], Email: j.clark.5@bham.ac.uk; Dr Zsombor Koszegi[3], Email: z.koszegi@bham.ac.uk; Prof Steve P Watson[1], Email: s.p.watson@bham.ac.uk

[1]Department of Cardiovascular Sciences, Level 1 IBR, School of Medical Sciences, College of Medicine and Health, University of Birmingham, Edgbaston, Birmingham B15 2TT, UK.

[2]Department of Metabolism and Systems Science, School of Medical Sciences, College of Medicine and Health, University of Birmingham, Edgbaston, Birmingham, B15 2TT, UK.

Supplementary Methods

Materials

Horm collagen was purchased from Takeda (London, UK). Rhodocytin was purified from the venom of *Calloselasma rhodostoma* as described.[1] Polyethylenimine (PEI) (PEI Max MW 40,000) was purchased from Polysciences (Pennsylvania, USA). TetraSpeck fluorescent beads were from Invitrogen, ThermoFisher Scientific (Paisley, UK). SNAP-Surface Alexa Fluor-647 was purchased from New England Biolabs (Hitchin, UK). Janelia Fluor-549 HaloTag Ligand was from Promega Corporation (Hampshire, UK). D-Luciferin sodium salt was from Cayman Chemical (Michigan, USA). Other reagents were obtained from Merck Life Science UK Limited.

Antibodies

Anti-GPVI HY101, and anti-mouse IgG Alexa Fluor 647 were purchased from Invitrogen, ThermoFisher Scientific (Paisley, UK). The generation of the CLEC-2 monoclonal antibody AYP1 has been reported.[2] AYP1 was produced in house using the generated hybridoma cell lines and purified using protein G affinity chromatography.

Construct generation

All constructs were sequenced for correctness.

GPVI: an untagged full length GPVI construct[3] was made by insertion of a stop codon TGA into a GPVI-eGFP construct downstream of the GPVI sequence and upstream of the eGFP sequence using the forward primer TCCGCTAGCGCTACCGGACTCAGATCCA and the reverse primer CGACTGCAGTCATGAACATAACCCGCGGCT.

SNAP-tag and HaloTag-GPVI: DNA coding for N-terminally tagged (extracellular) SNAP-tag and HaloTag-GPVI was obtained commercially from Twist Bioscience (San Francisco, USA). The DNA was subcloned into the mammalian expression vector pCI-neo (Promega Corporation, Hampshire, UK).

CLEC-2: DNA coding for untagged full length CLEC-2 was inserted into the pSF-CMV-puro-NH2-10HIS-Thr mammalian expression vector (Oxgene, Merck Life Science UK Limited).

SNAP-tag and HaloTag-CLEC-2: DNA coding for SNAP-tag was subcloned into the CLEC-2-pSF-CMV-puro-NH2-10HIS-Thr construct described above where the SNAP-tag was inserted at the C-terminal (extracellular) of CLEC-2. The N-terminal His_{10}-tag and thrombin cleavage site were removed by site-directed mutagenesis using the forward primer ATGCAGGATGAAGATGGATAC and the reverse primer CGCCATCGTGAGTACCTC. This was performed using Q5 site directed mutagenesis kit (New England Biolabs). DNA coding for C-terminally tagged (extracellular) HaloTag-CLEC-2 was obtained commercially from Twist Bioscience (San Francisco). The DNA was subcloned into the mammalian expression vector pCI-neo (Promega Corporation, Hampshire, UK).

SNAP-tag-CD86: DNA coding for N-terminal (extracellular) SNAP-tag-CD86 has been previously reported.[4]

Expression and purification of GPVI and CLEC-2 monovalent and multivalent nanobodies

The GPVI monovalent nanobody, Nb2 and CLEC-2 monovalent nanobody, Nb4 (also known as LUAS) were selected for cross-linking to generate di- and trivalent nanobody ligands. Nb2-2, Nb2-3, Nb4-2 and Nb4-3 were created using a short flexible $GGGGS_3$ linker between two or three copies of the original nanobody protein sequence. Generation of the plasmid DNA was outsourced to VIB Nanobody Core (Belgium). The monovalent and divalent ligands were expressed and purified from E. coli WK6 cells as described previously.[5-7] The trivalent ligands were expressed and purified from mammalian HEK293T cells as described previously.[7] For mono, di- and trivalent nanobodies nickel NTA beads were used. Purity was confirmed by SDS-PAGE.

Cell culture

Cell lines were purchased from American Type Culture Collection (ATCC). Chinese hamster ovary cells (CHO) were cultured at 37°C and 5% CO_2 in DMEM/F-12 (phenol red free) supplemented with 10% fetal bovine serum, 1% penicillin, 1% streptomycin and 1% glutamine. DT40 chicken B-cells were cultured at 37°C and 5% CO_2 in RPMI supplemented with 10% fetal bovine serum, 1% penicillin, 1% streptomycin, 1%

glutamine, 1% chicken serum and 50 µM 2β-mercaptoethanol. For transfection of CHO cells, PEI was used at a DNA to PEI ratio of 1 µg: 3 µl in non-supplemented DMEM/F-12. For transfection of DT40 cells, cells were electroporated in the presence of plasmid DNA in non-supplemented RMPI at 0.350kV and 0.500 F (GenePulser II, Bio-Rad).

Nuclear Factor of Activated T-cell (NFAT) assay

All GPVI and CLEC-2 constructs (details above) were used at 2 µg in combination with 15 µg NFAT-luciferase reporter DNA. For GPVI transfections, 2 µg FcRγ-chain was added. DT40 cells ($2x10^7$/transfection) were transfected with the construct DNA in serum-free RPMI by electroporation as described above. The following day cells were incubated with collagen (100 ng/ml, 1 µg/ml or 10 µg/ml), rhodocytin (1, 10 or 100 nM), positive controls PMA (50 ng/ml) and ionomycin (1 µM) or RPMI for 6 h at 37°C and frozen at -80°C. The next day, cells were harvested using luciferase harvest buffer (1M KH_2PO_4, 12.5% Triton X-100 and 1M dithiothreitol (DTT)) and added to luciferase assay buffer (1M KH_2PO_4, 0.1M $MgCl_2$, 0.1M ATP in ddH_2O) in a white, opaque 96-well plate. Luciferin substrate (50 ul at 1 mM) made in ddH_2O was added into the wells. Luciferase activity was measured with a Varioskan LUX Multimode Microplate Reader (Thermo Fisher Scientific) (counting time 10 sec per well). Transfection success of GPVI and CLEC-2 was assessed by flow cytometry as described in Supplementary Materials.

Flow cytometry to measure GPVI and CLEC-2 expression

To measure expression of the GPVI and CLEC-2 constructs (described above) used in NFAT assays on the surface of DT40 cells, a sample of each transfection was stained with HY101 anti-GPVI antibody (1.25 µg/ml) or AYP1 anti-CLEC-2 antibody (66 nM) followed by anti-mouse Alexa Fluor-647 secondary antibody staining (1:400). The samples were acquired and analysed in an Accuri C6 flow cytometer (BD Biosciences, USA). Cell populations were gated on cell size using forward scatter (FSC) vs side scatter (SSC) to distinguish them from electronic noise. The light scatter and fluorescent channel (FL4) were set to logarithmic gain and 10,000 events per sample were analysed. Data expressed as MFI (a.u) and histograms were made using FlowJo v10.0.7 (Eugene, OR). Statistical analysis was by a one-way ANOVA with a Bonferroni post-hoc test.

Single-particle tracking sample preparation

CHO cells were seeded in phenol red-free DMEM/F-12 at a density of $5x10^4$ cells/coverslip onto cleaned 25 mm coverslips (VWR, high precision, thickness No. 1.5H [0.170 ± 0.005 mm]). Prior to use coverslips were acid cleaned with 1M HCl followed by 100% ethanol and air-dried. The following day, cells were transiently transfected with PEI reagent in serum-free DMEM/F-12 (phenol red-free) according to manufacturer's instructions (PEI:DNA ratio = 3:1; 3 µg:1 µg) where 350 ng SNAP-tag GPVI/CLEC-2, 200 ng HaloTag GPVI/CLEC-2 or 500 ng SNAP-tag-CD86 DNA were used to achieve optimal receptor density. Empty pCI-neo vector was used to ensure total cDNA concentrations were consistent across all transfections, Cells were left to grow a further 24 h at 37°C/5% CO_2. Cells were labelled with 1 µM SNAP-Surface Alexa Fluor 647 (AF647, cell impermeable, New England Biolabs) and 1 µM HaloTag Janelia 549 (JF549, cell permeable, Promega) in complete culture medium for 20 min at 37 °C. Cells were washed three times with complete culture medium, allowing 10 min incubation between washes to remove non-specific labelling.

Single-particle tracking imaging

Single-particle tracking experiments were performed using total internal reflection fluorescence (TIRF) illumination on a custom system[8] (assembled by CAIRN Research) based on an Eclipse Ti2 microscope (Nikon) equipped with a 100x oil-immersion objective (SR HP APO TIRF NA 1.49, Nikon), 405, 488, 561, and 637 nm diode lasers (Coherent, Obis), an iLas2 TIRF illuminator (Gataca Systems), quadruple band excitation and dichroic filters, a quadruple beam splitter, 1.5x tube lens, four EMCCD cameras (iXon Ultra 897, Andor), hardware focus stabilisation, and a temperature-controlled enclosure. The sample and objective were maintained at 37 °C throughout the experiments. Coverslips were mounted in a microscopy chamber filled with HBSS, pH 7.5. Individual cells with comparable expression levels of the receptor constructs as indicated by their fluorescence intensity were selected for single-molecule analyses, resulting in similar densities within the compared groups. Multi-color single-molecule image sequences were acquired simultaneously on the two synchronized EMCCDs at a rate of one image every 30 ms for 12 sec. Following agonist addition, recordings made in the first 15 minutes were grouped and compared to those from 15-30 minutes to investigate the initial and sustained changes in distribution.

Single-particle tracking analysis

Automated single-particle detection and tracking were performed with the u-track software[9] and the obtained trajectories were further analysed using custom algorithms in MATLAB environment as previously described.[8,10] Image sequences from different channels were registered against each other, based on reference points taken with multi-color fluorescent beads (100 nm, TetraSpeck).[10] The inter-channel localisation precision after coordinate registration was ~20 nm. The time-averaged mean squared displacement (TAMSD) of individual trajectories was computed as previously described.[4,10] To obtain the diffusion coefficient (D), only trajectories lasting at least 100 frames were analysed. Trajectories were classified into four diffusion groups according to the diffusion parameters diffusion coefficient (D) and anomalous diffusion exponent (α). Molecules with $D < 0.01$ $\mu m^2 s^{-\alpha}$ were classified as immobile. Molecules classified under normal diffusion had $D \geq 0.01$ $\mu m^2 s^{-\alpha}$ and $0.75 \leq \alpha \leq 1.25$. Molecules with sub- and superdiffusion classifications had $D \geq 0.01$ $\mu m^2 s^{-\alpha}$ and $\alpha < 0.75$ or $\alpha > 1.25$, respectively.

The frequency and duration of GPVI-GPVI and CLEC-2-CLEC-2 interactions were estimated using previously described methods based on deconvolution of the distribution of single-molecule colocalisation times with the one expected for random colocalisations.[4] Trajectory segments were first linked to obtain continuous trajectories that are no longer interrupted by merging and splitting events. Then, for each particle in the SNAP-tag channel at frame f, all particles in the HaloTag channel falling within a defined search radius (150 nm) were identified as colocalising. If a colocalisation was also present at frame f + 1, the colocalisation was extended. The process was iterated until the last frame of the image sequence. These data were used to build a matrix containing information for each colocalisation (involved particles as well as the start and end frames). The observed colocalisation time corresponds to the duration of true interactions plus the duration of random colocalisations. Thus, the distribution of the observed colocalisation times can be seen as a convolution of the distribution of true interaction times and random colocalisation times. The distribution for random colocalisations was estimated in cells co-transfected with HaloTag-GPVI or CLEC-2 and monomeric SNAP-tag-CD86. To obtain the true colocalisation time, deconvolution with the Lucy−Richardson algorithm was performed.

Calculation of K_{on} and K_{off}

K_{on} and K_{off} values were calculated as reported before[4,8]. Briefly, particle loss due to photobleaching and tracking errors has been calculated based on simulations, which were designed to replicate experimental conditions (particle density, diffusion coefficient, and fluorophore bleaching rate) and a correction factor was used in the estimation of K_{off} values, and thus interaction times. K_{on} values were estimated separately at each frame, and the given values were obtained by averaging over the analysed frames.

Surface Plasmon Resonance binding studies

Surface plasmon resonance experiments were performed using a Biacore T200 instrument (GE Healthcare). Monomeric CLEC-2 (ECD 55-229) or GPVI (ECD 1-183 as produced previously[6]) was immobilised directly onto the CM5 chip using amine-coupling to the carboxylmethylated dextran-coated surface. Reference surfaces were blocked using 1M ethanolamine pH 8. Each concentration of analyte (CLEC-2 ECD or GPVI ECD) was run as follows; 120 sec injection, 300 sec dissociation and a 120 sec stabilisation period. All sensograms shown are double reference subtracted and at least two replicates were injected per cycle as well as experimental replicates of n=3. Experiments were performed at 25°C with a flow rate of 30 μL/min in HBS-EP running buffer (0.01 M HEPES pH 7.4, 0.15 M NaCl, 3 mM EDTA, 0.005% v/v surfactant P20). Multi-cycle kinetic assays were used with at least 5 concentration points between 0.1x and 10x the KD. Kinetic analysis was performed using the Biacore T200 Evaluation software using a global fitting to a 1:1 binding model and presented as mean ± standard error of mean (SEM).

Supplementary Figures

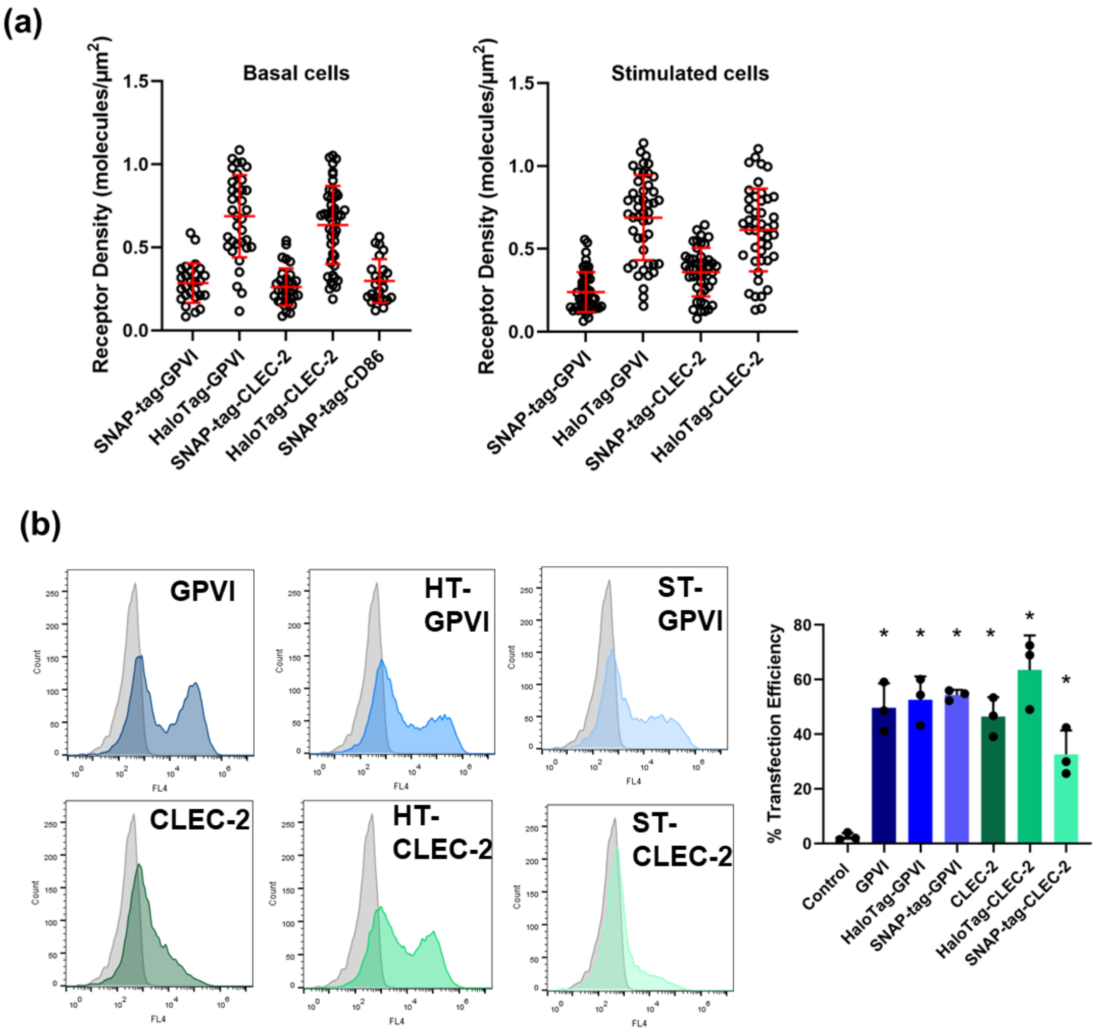

Supplementary Figure 1. Comparison of expression levels of GPVI and CLEC-2 constructs used in experiments in the study. (a) Receptor density (molecules/um^2) of SNAP- or Halo-tag GPVI or CLEC-2 and SNAP-tag-CD86 constructs expressed in CHO cells under basal and stimulatory conditions used in single particle tracking

studies (n=6 independent experiments). The average molecule density was calculated by counting and averaging the localised particles in frames 20 to 200 in the given area of the cell. (b) Expression of wild-type GPVI or CLEC-2 and SNAP- or Halo-tagged GPVI and CLEC-2 in DT40 chicken B-cells used in the NFAT assay, measured by flow cytometry using anti-GPVI HY101 (1.25 µg/ml) or anti-CLEC-2 AYP1 antibody (66 nM) with anti-mouse Alexa Fluor-647 secondary staining. Grey histograms show non-specific secondary staining alone. Flow cytometry data presented as % transfection efficiency of the GPVI and CLEC-2 constructs showing the percentage of positive cells. Significance was measured with a one-way ANOVA with a Bonferroni *post-hoc* test where $P \leq 0.05$. Data presented as mean±SD (n=3 independent experiments).

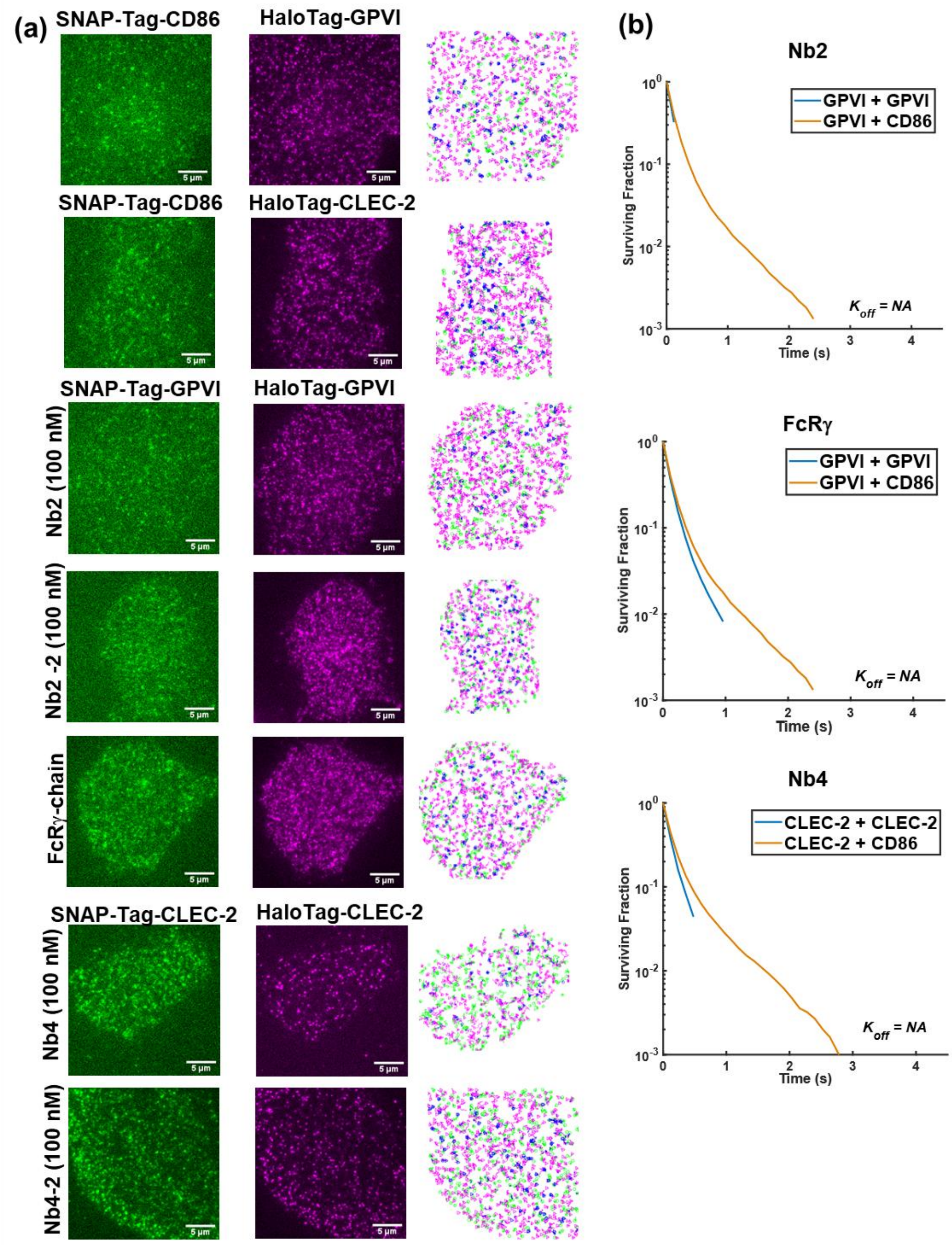

Supplementary Figure 2. Single-particle tracking of GPVI and CLEC-2. (a-b) Total internal reflection fluorescence (TIRF) microscopy was used to image live CHO cells for 400 frames (~12 seconds) expressing the receptor combinations: Halotag-GPVI + SNAP-tag-GPVI, Halotag-CLEC-2 + SNAP-tag-CLEC-2, Halotag-GPVI + SNAP-tag-CD86 and Halotag-CLEC-2 + SNAP-tag-CD86. CD86 was used as a non-interacting control protein. Generated videos were tracked to generate trajectories and analysed as described in the methods section. (a) Two-colour single particle tracking was used to analyse GPVI-GPVI and CLEC-2-CLEC-2 interactions under basal conditions and following ligand addition. Representative images of CHO cells imaged using TIRF microscopy under basal conditions and following stimulation with the labelled treatments, where green = SNAP-tag-receptors labelled with Alexa Fluor-647 and magenta = Halotag-receptors labelled with Janelia Fluor-549 and the corresponding dual channel tracking analysis showing the trajectories of SNAP- and Halo-tag-receptors. Blue shows colocalisation of the two channels (scale bar: 5 mm for all images). (b) Interaction survival curves for GPVI-GPVI or CLEC-2-CLEC-2 (blue) and GPVI/CLEC-2-CD86 (orange) interactions following monovalent nanobody (Nb2 or Nb4, 100 nM) stimulation and FcRγ-chain expression (GPVI). No true interactions could be detected following deconvolution with SNAP-tag-CD86 (estimate random colocalisations) and no kinetic parameters (K_{on} and K_{off}) could be determined (NA).

References

1. Eble, J. A., Beermann, B., Hinz, H. J. & Schmidt-Hederich, A. alpha 2beta 1 integrin is not recognized by rhodocytin but is the specific, high affinity target of rhodocetin, an RGD-independent disintegrin and potent inhibitor of cell adhesion to collagen. *J Biol Chem* **276**, 12274-12284, doi:10.1074/jbc.M009338200 (2001).
2. Gitz, E. *et al.* CLEC-2 expression is maintained on activated platelets and on platelet microparticles. *Blood* **124**, 2262-2270, doi:10.1182/blood-2014-05-572818 (2014).
3. Clark, J. C. *et al.* Evidence that GPVI is Expressed as a Mixture of Monomers and Dimers, and that the D2 Domain is not Essential for GPVI Activation. *Thromb Haemost* **121**, 1435-1447, doi:10.1055/a-1401-5014 (2021).
4. Sungkaworn, T. *et al.* Single-molecule imaging reveals receptor-G protein interactions at cell surface hot spots. *Nature* **550**, 543-547, doi:10.1038/nature24264 (2017).
5. Clark, J. C. *et al.* Divalent nanobodies to platelet CLEC-2 can serve as agonists or antagonists. *Commun Biol* **6**, 376, doi:10.1038/s42003-023-04766-6 (2023).
6. Slater, A. *et al.* Structural characterization of a novel GPVI-nanobody complex reveals a biologically active domain-swapped GPVI dimer. *Blood* **137**, 3443-3453, doi:10.1182/blood.2020009440 (2021).
7. Martin, E. M. *et al.* Trivalent nanobody-based ligands mediate powerful activation of GPVI, CLEC-2, and PEAR1 in human platelets whereas FcγRIIA requires a tetravalent ligand. *J Thromb Haemost* **22**, 271-285, doi:10.1016/j.jtha.2023.09.026 (2024).
8. Grimes, J. *et al.* Plasma membrane preassociation drives β-arrestin coupling to receptors and activation. *Cell* **186**, 2238-2255.e2220, doi:10.1016/j.cell.2023.04.018 (2023).

9. Jaqaman, K. *et al.* Robust single-particle tracking in live-cell time-lapse sequences. *Nat Methods* **5**, 695-702, doi:10.1038/nmeth.1237 (2008).
10. Calebiro, D. *et al.* Single-molecule analysis of fluorescently labeled G-protein-coupled receptors reveals complexes with distinct dynamics and organization. *Proc Natl Acad Sci U S A* **110**, 743-748, doi:10.1073/pnas.1205798110 (2013).
