# Supplementary figures and images for: Receptor homodimerization significantly prolongs the lifetime of ligand-induced cross-linking of CLEC-2 but not GPVI
Source: Blood Vessel Thromb Hemost. 2026 Mar 19;3(2):100160. doi: 10.1016/j.bvth.2026.100160 (PMC13181292; doi:10.1016/j.bvth.2026.100160)

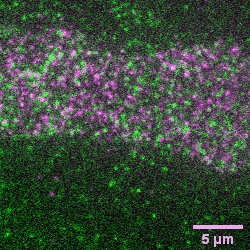

Supplement: Supplemental Video 1 left side [file BVTH_VTH-2025-000487-mmc2.gif]

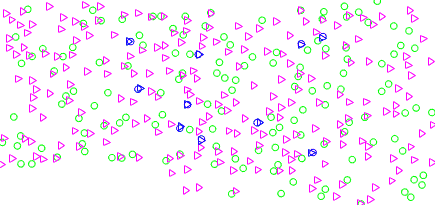

Supplement: Supplemental Video 1 right side [file BVTH_VTH-2025-000487-mmc3.gif]

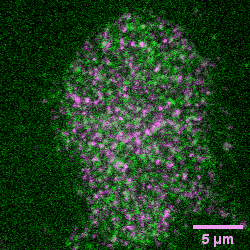

Supplement: Supplemental Video 2 left side [file BVTH_VTH-2025-000487-mmc4.gif]

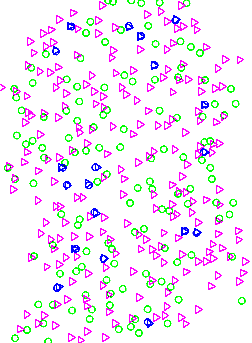

Supplement: Supplemental Video 2 right side [file BVTH_VTH-2025-000487-mmc5.gif]

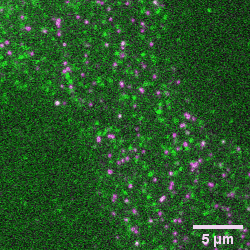

Supplement: Supplemental Video 3 left side [file BVTH_VTH-2025-000487-mmc6.gif]

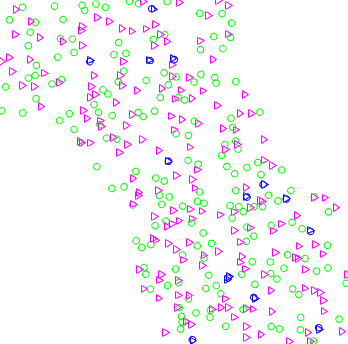

Supplement: Supplemental Video 3 right side [file BVTH_VTH-2025-000487-mmc7.gif]

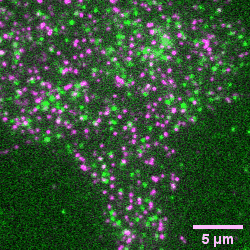

Supplement: Supplemental Video 4 left side [file BVTH_VTH-2025-000487-mmc8.gif]

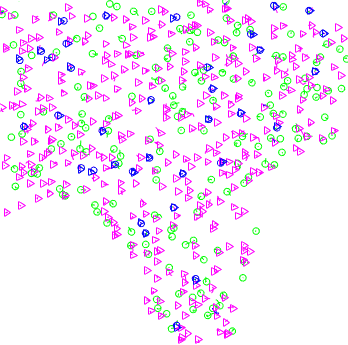

Supplement: Supplemental Video 4 right side [file BVTH_VTH-2025-000487-mmc9.gif]

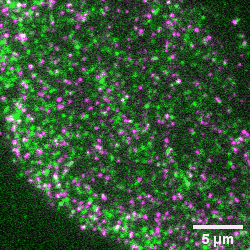

Supplement: Supplemental Video 5 left side [file BVTH_VTH-2025-000487-mmc10.gif]

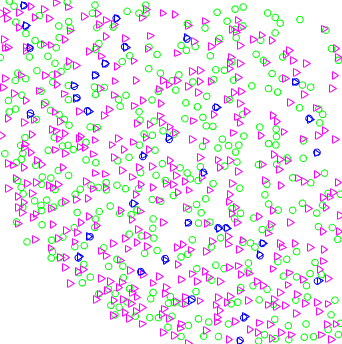

Supplement: Supplemental Video 5 right side [file BVTH_VTH-2025-000487-mmc11.gif]

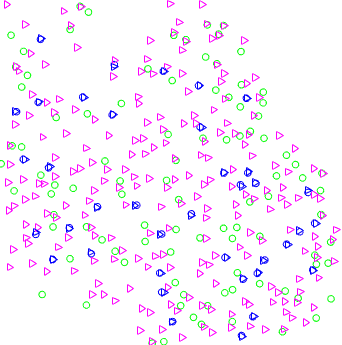

Supplement: Supplemental Video 6 left side [file BVTH_VTH-2025-000487-mmc12.gif]

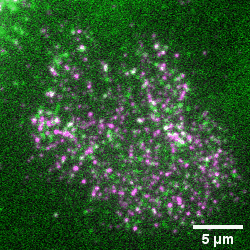

Supplement: Supplemental Video 6 right side [file BVTH_VTH-2025-000487-mmc13.gif]
